# Analysis of the Volatile and Enantiomeric Compounds Emitted by *Plumeria rubra* L. Flowers Using HS-SPME–GC

**DOI:** 10.3390/plants13172367

**Published:** 2024-08-25

**Authors:** James Calva, Jhoyce Celi, Ángel Benítez

**Affiliations:** 1 Departamento de Química, Universidad Técnica Particular de Loja, Loja 1101608, Ecuador; 2 Carrera de Bioquímica y Farmacia, Universidad Técnica Particular de Loja, Loja 1101608, Ecuador; jhceli@utpl.edu.ec; 3 Biodiversidad de Ecosistemas Tropicales-BIETROP, Herbario HUTPL, Departamento de Ciencias Biológicas y Agropecuarias, Universidad Técnica Particular de Loja (UTPL), San Cayetano s/n, Loja 1101608, Ecuador; arbenitez@utpl.edu.ec

**Keywords:** *Plumeria rubra* L., chemical analysis, enantiomeric analysis, HS-SPME, GC–MS

## Abstract

The volatile components emitted by fresh aromatic flowers of *Plumeria rubra* L., harvested in southern Ecuador during three different months were determined to evaluate the fluctuation of secondary metabolites. The volatile compounds were analyzed using headspace solid-phase microextraction (HS-SPME) followed by gas chromatography coupled to mass spectrometry (GC–MS) and a flame ionization detector (GC–FID) using two types of columns: a non-polar (DB-5ms) and polar column (HP-INNOWax). The principal chemical groups were hydrocarbon sesquiterpenes (43.5%; 40.0%), oxygenated sesquiterpenes (23.4%; 26.4%), oxygenated monoterpenes (14.0%; 11.2%), and hydrocarbon monoterpenes (12.7%; 9.3%). The most representative constituents were (E,E)-*α*-Farnesene (40.9–41.2%; 38.5–50.6%), (E)-nerolidol (21.4–32.6%; 23.2–33.0%), (E)-*β*-ocimene (4.2–12.5%; 4.5–9.1%), (Z)-dihydro-apofarnesol (6.5–9.9%; 7.6–8.6%), linalool (5.6–8.3%; 3.3–7.8%), and perillene (3.1–5.9%; 3.0–3.2%) in DB-5ms and HP-INNOWax, respectively. Finally, we reported for the first time the enantiomeric distribution of *P. rubra* flowers, where the enantiomers (1R,5R)-(+)-α-pinene, (S)-(−)-limonene, (S)-(+)-Linalool, and (1S,2R,6R,7R,8R)-(+)-α-copaene were present as enantiomerically pure substances, whereas (S)-(+)-(E)-Nerolidol and (R)-(+)-(E)-Nerolidol were observed as scalemic mixtures. This study provides the first comprehensive and comparative aroma profile of *Plumeria rubra* cultivated in southern Ecuador and gave us a clue to the variability of *P. rubra* chemotypes depending on the harvesting time, which could be used for future quality control or applications in phytopharmaceutical and food industries.

## 1. Introduction

Plants have been used for medicinal purposes by humans since ancient times, and their properties have contributed to the elimination of diseases and thus to survival [1]. According to WHO estimates, about 80% of the population uses herbal medicine for primary health care despite the growing technology of organic synthesis [2]. Apocynaceae, a family of flowering plants, belongs to the order Gentianales. It is one of the ten largest angiosperm families globally [3,4,5], consisting of approximately 366 genera and around 5100 species [6,7,8]. The family is divided into five subfamilies: Rauvolfioideae, Apocynoideae, Periplocoideae, Secamonoideae, and Asclepiadoideae. Apocynaceae has a widespread distribution, with members native to various regions, including Europe, Asia, Africa, Australia, and the Americas [8,9,10].

Ecuador, despite having an area of 283,561 km^2^, is characterized by a diversity of ecosystems with different microclimates and habitats [11]. Thus, due to its high biological and cultural diversity, it has become one of the countries with great potential in traditional medicine [12]. In this context, the genus *Plumeria* consists of many species distributed all over the world, 11 of which grow in tropical and subtropical regions [13]. Both essential oil and the aromatic components of the flowers of many of these species are used in perfumery, cosmetics, and aromatherapy [14]. The most popular species are *Plumeria obtusa* L., *Plumeria alba*, and *Plumeria rubra* L. [13].

*Plumeria rubra* L. is commonly known as “flor de mayo” and belongs to the Apocynaceae family and is native to Mexico [15]. However, due to its easy propagation by cutting, it has spread throughout the world, especially in warm regions such as Hawaii, where it is cultivated in abundance [16]. It grows as a small tree that can reach a height of two to eight meters [13]. In terms of medicinal use, it is reported that the decoction of *P. rubra* is traditionally used to treat asthma, constipation, to stimulate menstruation, and to reduce fever [17]. It is important to note that this species, which does not have a nectary, is pollinated by insects through floral mimicry [18]. In our country, it can be found in provinces such as Chimborazo, Los Ríos, El Oro, Manabí, Guayas, Esmeraldas, Imbabura, and the Galapagos Islands [19].

Chemical characterization of the secondary metabolites of *Plumeria obtusa*, plumieridin A, plumieridine, 1 *α*-plumieride, 15-demethylplumieride, rel-(3R,30 S,4R,40 S)-3,30,4,40-tetrahydro-6,60-dimethoxy [3,30-bi-2H-benzopyran]-4,40-diol, glochiflavanoside B, oleanolic acid, and methyl coumarate have been identified [20]. Also isolated from the stem bark of *P. rubra* were the compounds 1-(*p*-hydroxyphenyl) propan-1-one, isoplumericin, plumericin, dihydroplumericin, alamcin, fulvoplumerin, ala-mandine, plumieride, *p*-E-coumaric acid, 2,6-dimethoxy-*p*-benzoquinone, scopoletin, cycloart-25-en-3β,24-diol, 2,4,6-trimethoxyaniline, ajunolic acid, ursolic acid, oleanolic acid, β-amyrin acetate, betulinic acid, lupeol and its acetate, 2,3-dihydroxypropyl octacosanoate, and β-sitosterol glycoside [21].

In other countries, for example, Nigeria, the volatile compounds from *P. rubra* were (E)-non-2-en-1-ol (15.7%), limonene (10.8%), phenylacetaldehyde (9.0%), n-tetradecanal (8.8%), *γ*-elemene (6.5%), and (E,E)-*α*-farnesene (6.1%) [17]. In addition, in India, the compounds benzyl benzoate (22.3%, 7.9%), geraniol (trace, 17.2%), (E,E)-geranyl linalool (9.4%, 0.2%), tricosane (8.3%, 1.1%), linalool (0.1%, 8.0%), nonadecane (7.0%, 3.8%), (E)-nerolidol (7.0%, 5.5%), and pentacosane (4.4%, 0.3%) were reported in both the essential oil of the flowers and the volatile vapor extract, respectively [14].

A gas chromatograph coupled to a mass spectrometer (GC–MS) was used to identify the aromatic compounds of the species [22] and extracted using headspace solid-phase microextraction (HS-SPME), which is currently quite dominant due to its simplicity, absence of solvents, high sensitivity, and low cost [23]. This method is based on a fiber coated with one or more extraction polymers, which removes the analytes from the sample by adsorption to be subsequently introduced into the GC–MS system for thermal desorption and analysis [24].

The aim of this research was to determine the chemical composition and report, for the first time, the enantiomeric distribution of some terpenes emitted by *P. rubra* flowers that provides its characteristics such as its odor or therapeutic properties. In this way, it will lay the foundation for future research and, at the same time, contribute to the knowledge of new techniques for extracting compounds, since the technique to be tested (HS-SPME) has not been studied much in this field, although it has advantages over other common methods such as steam distillation or hydrodistillation, such as the time required and the absence of solvents for extracting compounds [25].

## 2. Results

### 2.1. Chemical Composition

**Figure 1:**
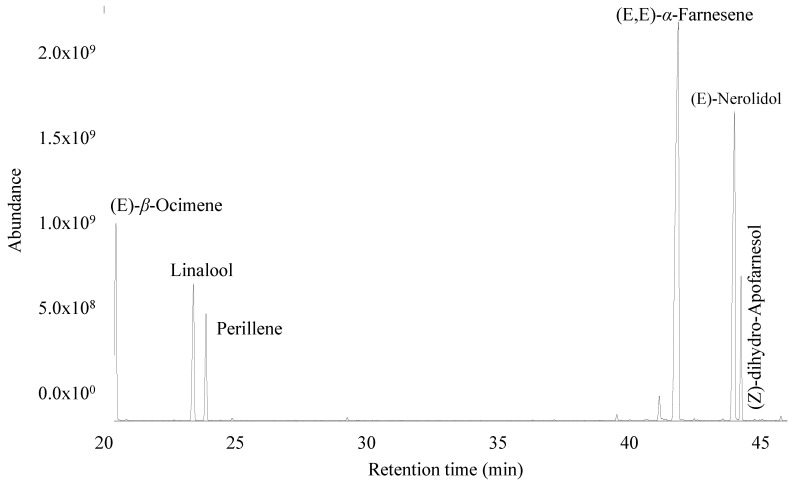
Gas chromatogram of the *Plumeria rubra* L. flowers, obtained using DB-5ms column.

**Figure 2:**
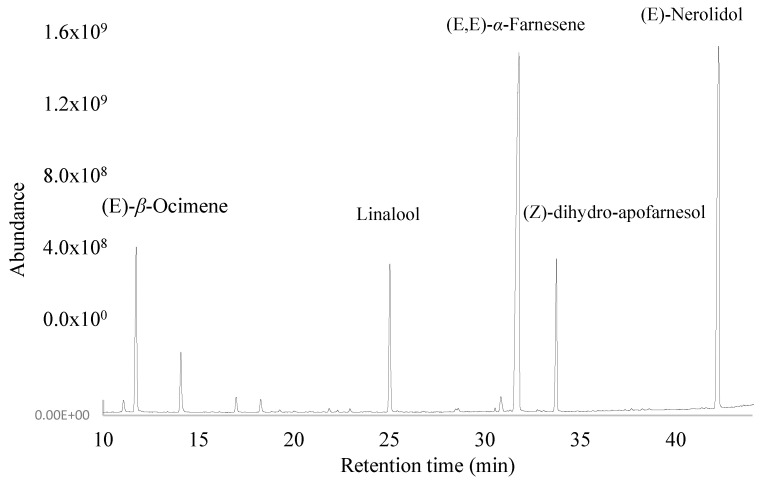
Gas chromatogram of the *Plumeria rubra* L. flowers, obtained using HP-INNOWax column.

A total of 59 and 53 volatile compounds in the flowers of the species *Plumeria rubra* L. were determined in three different months of collection in DB-5ms and HPINNOWax columns, respectively, and arranged according to the order of elution (Table 1 and Table 2, Figure 1 and Figure 2).

A total of 59 compounds were identified in the three months; the first extraction (March) was composed mainly of hydrocarbon sesquiterpenes (43.12%), followed by oxygenated sesquiterpenes (23.38%), oxygenated monoterpenes (14.03%), and hydrocarbon monoterpenes (12.73%). The most representative compounds were (E,E)-*α*-farnesene (41.64%), (E)-nerolidol (22.98%), (E)-*β*-ocimene (12.51%), linalool (8.28%), (Z)-dihydro-apofarnesol (6.46%), and perillene (5.48%).

The second extraction (May) was composed mainly of hydrocarbon sesquiterpenes (42.95%), followed by oxygen sesquiterpenes (21.89%), oxygen monoterpenes (17.28%), and hydrocarbon monoterpenes (11.04%). The majority of the compounds were (E,E)-*α*-farnesene (40.87%), (E)-nerolidol (21.40%), (E)-*β*-ocimene (10.82%), linalool (11.05%), (Z)-dihydro-apofarnesol (6.44%), and perillene (5.85%).

In the third extraction (July), the hydrocarbon sesquiterpenes were the most abundant (42.53%), followed by oxygen sesquiterpenes (32.95%), oxygen monoterpenes (9.51%), and hydrocarbon monoterpenes (4.21%). The majority of the compounds were (E,E)-*α*-farnesene (41.15%), (E)-nerolidol (32.55%), (E)-*β*-ocimene (4.16%), linalool (5.92%), (Z)-dihydro-apofarnesol (9.89%), and perillene (3.09%).

Using the polar column (HP-INNOWax), a total of 53 components were identified; in the first extraction (March), the main groups of compounds were hydrocarbon sesquiterpenes (40%), followed by oxygen sesquiterpenes (26.4%), oxygen monoterpenes (11.2%), and hydrocarbon monoterpenes (9.3%). The most representative compounds were (E,E)-*α*-farnesene (38.48%), (E)-nerolidol (26.28%), (E)-*β*-ocimene (9.06%), linalool (7.80%), (Z)-dihydro-apofarnesol (7.5%), and perillene (3.19%).

In the second extraction (May), the main groups were hydrocarbon sesquiterpenes (39.75%), followed by oxygen sesquiterpenes (33.12%), alcohols (8.83%), oxygen monoterpenes (7.30%), and hydrocarbon monoterpenes (4.96%). The main compounds were (E,E)-*α*-farnesene (38.00%), (E)-nerolidol (33.02%), (E)-*β*-ocimene (4.96%), linalool (4.93%), (Z)-dihydro-apofarnesol (7.70%), and perillene (5.85%).

Finally, in the third extraction (July), the main groups were hydrocarbon sesquiterpenes (52.57%), followed by oxygen sesquiterpenes (23.81%), alcohols (9.12%), oxygen monoterpenes (6.45%), and hydrocarbon monoterpenes (5.92%). The most representative compounds were (E,E)-*α*-Farnesene (50.59%), (E)-Nerolidol (23.16%), (E)-*β*-Ocimene (5.71%), Linalool (3.27%), (Z)-dihydro-apofarnesol (8.62%), and Perillene (3.09%).

In order to complement the information on the main compounds identified in this study, the biological activities related to therapeutic effects on the organism is presented in Table 3.

### 2.2. Enantiomeric Distribution

**Figure 3:**
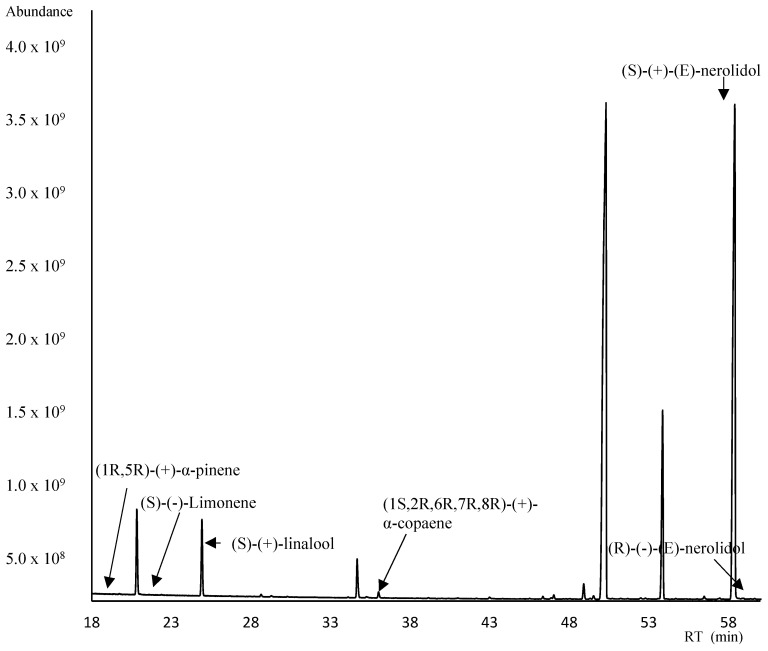
Enantiomeric analysis of the *Plumeria rubra* L. flowers using the β-cyclodextrin column.

The enantioselective analysis permitted the identification of three enantiomerically pure compounds in *P. rubra* flowers. They were (1R,5R)-(+)-α-pinene; (S)-(−)-limonene; (S)-(+)-Linalool; (1S,2R,6R,7R,8R)-(+)-α-copaene; and (E)-nerolidol. Detailed results of the enantioselective analysis are given in Table 4, Figure 3.

**Figure 4:**
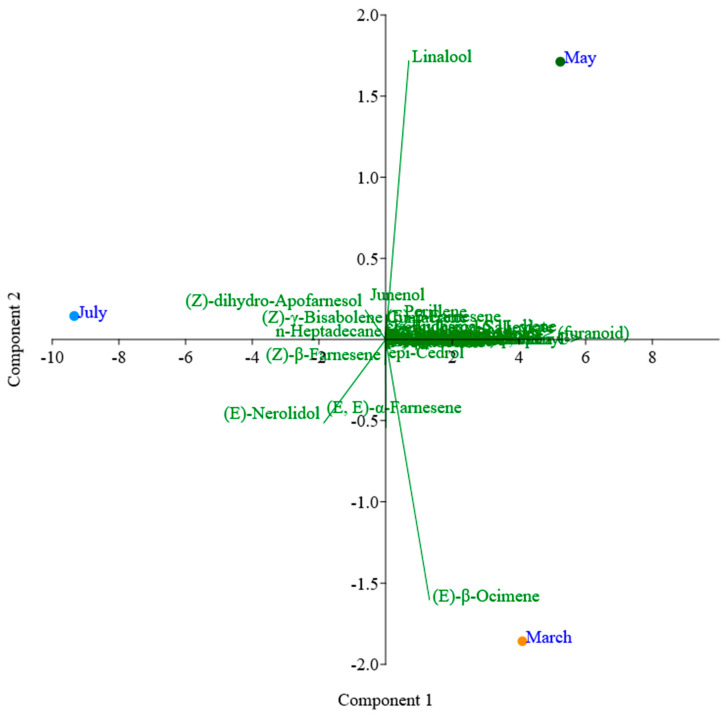
PCA analysis of the *Plumeria rubra* L. flowers using the DB-5ms column.

The PCA of the chemical composition of the volatile compounds of flowers using the DB-5ms column (Figure 4) showed different compounds found in the different months of collection. After the PCA analysis, it was possible to determine the dispersion of the chemical composition obtained after chromatographic analysis on the DB-5ms column. The first component accounted for 95.41% of the total variance in the data set, characterized by the compounds (Z)-*β*-farnesene, cedrol, *δ*-decalactone, perillene, and junenol, among others, while the second component accounted for 4.58% of the variance, characterized by the compounds (E)-*β*-ocimene, (E,E)-*α*-farnesene, (E)-nerolidol, and linalool, among others. In terms of similarity, the months of March and May showed greater variability than July.

**Figure 5:**
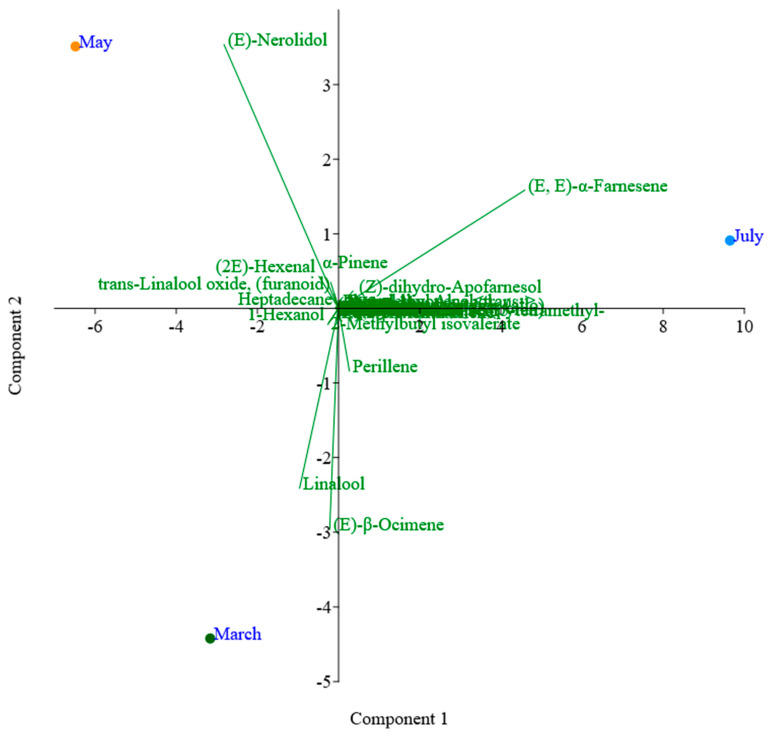
PCA analysis of the *Plumeria rubra* L. flowers using the HP-INNOWax column.

In the PCA analysis of HP-INNOWax column, the first component accounted for 85.24% of the total variance in the data set, characterized by the compounds 1-hexanol, (E)-tridecen-1-ol, (2E)-hexenal, hexanal, and (E,E)-*α*-farnesene, among others, while the second component accounted for 15.75% of the variance in the data set, characterized by the compounds linalool, (E)-*β*-ocimene, perillene, and (E)-nerolidol. In terms of similarity, the months of March and May showed a higher variance (Figure 5).

## 3. Discussion

The use of orthogonal columns confirms the identification of a greater variety of compounds [57]. According to the results, approximately 53–59 different compounds were identified in both columns; only 14 of them were found in both phases, including the main constituents. This affinity of the stationary phase results in a different elution order. In the non-polar column, the stationary phase (5% phenylpolydimethylsiloxane) presents an affinity for the less polar compounds and will therefore retain them until the end of the chromatographic run, eluting the non-polar compounds first. This contrasts the stationary phase (polyethylene glycol) of the polar column, which has a higher affinity for the polar compounds, eluting the polar compound first [57].

In this study, in *P. rubra* flowers, the main volatile compounds identified were (E,E)-*α*-Farnesene (40.9–41.2%; 38.5–50.6%), (E)-nerolidol (21.4–32.6%; 23.2–33.0%), (E)-*β*-ocimene (4.2–12.5%; 4.5–9.1%), (Z)-dihydro-apofarnesol (6.5–9.9%; 7.6–8.6%), linalool (5.6–8.3%; 3.3–7.8%), and perillene (3.1–5.9%; 3.0–3.2%) in DB-5ms and HP-INNOWax, respectively. Previous studies reported (E,E)-*α*-farnesene, (E)-nerolidol, and linalool, similar to the studies of ElZanaty et al. (2022) [58], who identified methyl dihydroepi-jasmonate (35.41%), linalool (14.31%), and methyl jasmonate (11.99%) as main constituents. Goswami et al. (2016) [14] reported benzyl salicylate (26.7%), benzyl benzoate (22.3%), (E,E)-geranyl linalool (9.4%), tricosane (8.3%), linalool (0.1%), nonadecane (7.0%), (E)-nerolidol (7.0%), and pentacosane (4.4%) as main constituents. And finally, in the study by Lawal et al. (2015) [17], they identified limonene (10.8%), phenylacetaldehyde (9.0%), n-tetradecanal (8.8%), *γ*-elemene (6.5%), and (E,E)-*α*-farnesene (6.1%) as main constituents. Our results show a relative quantitative and qualitative variation in the compounds found compared to those reported in these studies on *P. rubra* flowers, mainly because the EOs were extracted using hydrodistillation.

The chemical compound (E)-*β*-ocimene, one of the main compounds reported in our study, was also mentioned in the study by Barreto et al. (2014) [18], but as a minority. For the majority of reported compounds, including (Z)-dihydro-apofarnesol and perillene, no reports were found, even in other analyses performed on species of the same genus.

In contrast, other studies such as that of Lui et al. (2012) [59], indicate that the major components of the EO of *P. rubra* flowers obtained using hydrodistillation were n-hexadecanoic acid (35.8%) and n-tetradecanoic acid (11.2%). Meanwhile, Omata et al. (1991) [60] reported trans-phenylacetaldehyde, trans-farnesol, 8-phenylethyl alcohol, geraniol, α-terpineol, neral, and geranial as the main compounds. In addition, Tohar et al. (2006) [15], using hydrodistillation, found non-terpenic esters (benzyl salicylate, benzyl benzoate, and 2-phenylethyl benzoate) and alkanoic acids.

The findings confirm that terpenes, including sesquiterpenes and monoterpenes, were the prominent chemical group and main contributors to the composition and fragrance of *P. rubra* flowers. According to Gong et al. (2019) [61], floral aromas are dominated by fatty acid derivatives, terpenoids (mono or sesquiterpenes), and phenylpropanoids/benzenoids. In addition, a study using HS-SPME by Baéz et al. (2012) [62] in *P. tuberculate*, found oxygenated monoterpenes (79.6%), oxygenated sesquiterpenes (8.4%), hydrocarbons (7.6%), and benzenoid esters (2.6%) as main groups; this result shows similarities to our study.

Chemical variability is associated with primary causal factors, including genetic differences between plants; the growing environment (humidity, sunlight, soil, and nutrient bioavailability); the life cycle stage of the plants, as their composition may vary at early or late stages; biological interactions with animal species such as pollinators [61]; and the time between collection and extraction, as a longer interval may lead to a decrease or change in the composition of the volatile analytes present [63].

For the first time, as a contribution of new knowledge, it is reported the enantioseparation of some terpenes, and for one of them, i.e., E-nerolidol, the presence of the distomer R has been detected, although at a very low level (0.14%), in *P. rubra* flowers. It is well known that enantiomers are chiral compounds with identical physical and chemical properties, except for their optical activity, and can also exhibit different biological effects, and some species use their stereochemical properties for communication [64].

The HS-SPME analysis differs from the hydrodistillation used in the studies by Barreto et al. (2014) [18], ElZanaty et al. (2022) [58], and Goswami et al. (2016) [14]. In addition, both techniques aim to extract volatile compounds, with the difference that hydrodistillation is applied more to essential oils and extracts a wide range of volatile compounds, some of which may be difficult to capture with HS-SPME [64]. However, considering that our study works with flowers of a cultivated species, the sample size is limited, since the application of HS-SPME is more selective and faster due to its automated facility, short analysis time, and was used with different types of biological samples [25] and a small amount of sample [65].

## 4. Materials and Methods

### 4.1. Plant Material

**Figure 6:**
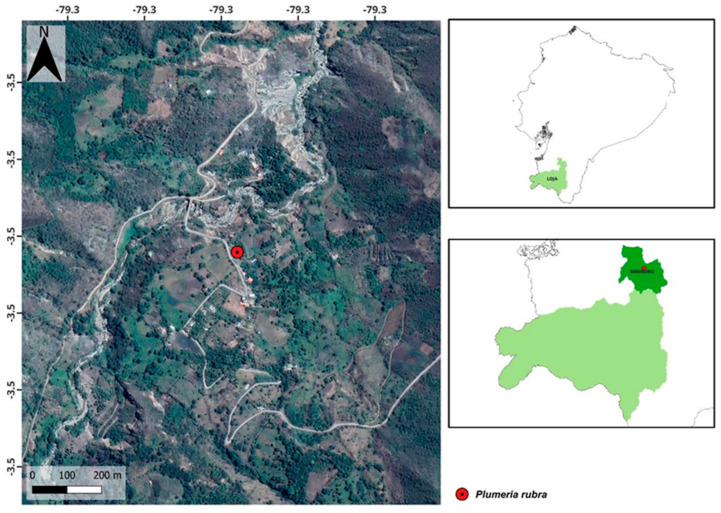
Map of the collection of *Plumeria rubra* L. flowers from southern Ecuador.

*Plumeria rubra* L. fresh flowers were collected during the morning in the sector of San Antonio, San Pablo de Tenta, Saraguro, Loja, with the coordinates 3°30′59.1″ S 79°17′36.9″ W (Figure 6). The collection was authorized by the Ministry of Environment, Water and Ecological Transition (MAATE) with authorization code MAATE-ARSFC-2022-2839. The authenticity of the species was verified by Ing. Jorge Armijos, curator of the HUTPL herbarium, who registered with voucher number 14,778.

### 4.2. Selection and Preparation of Plant Material

The flowers were harvested and only those in good condition (free of dried or wilted flowers) were selected; their petals were cut into small pieces to release more volatile compounds. A total of 5 g was weighed using an analytical balance and placed in a 100 mL bottle with a headspace for introducing the SPME fiber.

### 4.3. Extraction of Compounds Using Solid-Phase Microextraction

The headspace solid-phase microextraction (HS-SPME) technique described in [24,66] with some modifications was used for the extraction of the volatile compounds. Prior to use, the fiber was conditioned in the GC injector at 250 °C for 0.5 h. Five grams of the flower was then placed in a 100 mL sample vial, which was sealed with septum-type caps Supelco (Bellefonte, USA). The vial was then heated to 45 °C for 15 min. The septum was then pierced with a solid-phase microextraction (SPME) needle and a DVB/CAR/PDMS (divinylbenzene/carboxene/polydimethylsiloxane) fiber was extended through the needle. The fiber was exposed to the headspace above the sample for 15 min to capture volatile compounds. After an optimized extraction time of 15 min, the fiber was retracted into the needle. Finally, the needle was removed from the septum and inserted directly into the GC injection port. Desorption of the analytes from the fiber coating was achieved by heating the fiber at 250 °C for 5 min in the splitless injection mode.

### 4.4. Analysis of Volatile Compounds GC–MS

Volatile compounds extracted from *P. rubra* species were analyzed using a Thermo Scientific model TRACE 1310 gas chromatograph (Waltham, MA, USA) coupled to an ISQ7000 mass spectrometer (Bartlesville, OK, USA) equipped with a Chromeleon 7.0 Chromatography Studio data system. Spectra were recorded in full scan mode, in the mass range of 30 to 350 amu, at a scan rate of 0.2 scans/s.

The separation of the compounds was carried out on two capillary columns, an apolar stationary phase DB-5ms (5% phenyl 95% polydimethylsiloxane, 30 m × 0.25 mm i.d., film thickness 0.25 µm) and polar stationary phase HP-INNOWax (polyethylene glycol, 30 m × 0.25 mm i.d., film thickness 0.25 µm) from J & W Scientific (Folsom, CA, USA) [67]. Helium (99.999% purity) (Indura, Guayaquil, Ecuador) was used as carrier gas at a constant flow rate of 1 mL/min [68]. The injection mode was split (10:1) with a temperature of 250 °C at the injector. The ion source temperature was set to 230 °C and 150 °C quadrupled. The chromatography oven was programmed from 40 to 150 °C (3 °C/min), then to 180 °C (5 °C/min) for 5 min, and finally to 230 °C (7 °C/min) for a total run time of 67 min.

### 4.5. Analysis of Volatile Compounds GC–FID

For quantitative analysis, the same equipment was used as for the GC–MS analysis, except that it was coupled to a flame ionization detector (GC–FID). Samples were injected under the same conditions as described above. The injector temperature was 270 °C and the injector gas mixture was UHP hydrogen (30 mL/min), zero grade air (300 mL/min), and UHP nitrogen (45 mL/min) [69] using a Parker gas generator hydrogen generator (UK-UK). The content (%) of each identified oil component was calculated as the % of the area of the corresponding peak in the gas chromatography–flame ionization detector (GC–FID) chromatogram compared to the sum of the areas of all identified peaks. No correction factor was applied.

### 4.6. Compound Identification

The components of the flowers were identified by comparing the linear retention indices (LRIs), calculated according to Van den Dool and Kratz (70), and mass spectra with data from the literature. A mixture of n-alkanes C_9_-C_24_ (ChemService, West Chester, PA, USA) [70] was used. For compounds analyzed on the DB-5ms column, peaks were identified by comparing mass spectra with their LRIs using the ADAMS book [26]. For the HP-INNOWax column, the NIST library (NIST Libraries, National Institute of Standards and Technology, Gaithersburg, MD, USA) was used [71]. The compound was considered identified if the calculated retention index did not differ by ±25 from the reference values [22].

### 4.7. Enantiomeric Analysis

The chromatographic conditions to achieve the enantioseparation of some chiral terpenes present in *P*. *rubra* flowers involved the use of a chiral capillary column MEGA-DEX-DAC based on 2,3-diacetyl-6-tert-butyldimethylsilyl-β-cyclodextrin (25 m, 0.25 mm film thickness, 0.25 µm, purchased from MEGA S.r.l. (Legnano, MI, Italy)). The temperature programme for the gas chromatography (GC) oven was as follows: an initial temperature of 50 °C for 1 min, followed by a gradient increase of 2 °C per minute until 220 °C; finally, it was maintained for 10 min. In addition, the enantiomers were identified and compared by their MS spectrum, linear retention indices from the bibliography, and by the injection of enantiomerically pure standards (Sigma–Aldrich, St. Louis, MO). Using the formula originally proposed by van Den Dool and Kratz, we calculated linear (arithmetic) retention indices [68,72].

### 4.8. Statistical Analysis

The analysis of volatile compounds in relation to the harvest time and the type of column used was carried out using principal component analysis (PCA), a multivariate analysis technique that makes it possible to visualize the similarities or differences between a group of data, which in this study refers to the compounds found [73]. All these analyses were carried out using the statistical software PAST 4.10 [74].

## 5. Conclusions

In this study, we reported for the first time the volatile composition of *Plumeria rubra* L. flowers using a HS-SPME–GC from Ecuador, and it was found that there was a significant difference between collection times of the species. The chemical groups more representative were hydrocarbon sesquiterpenes (43.48%), followed by oxygen sesquiterpenes (26.92%), oxygen monoterpenes (10.95%), alcohols (8.25%), and hydrocarbon monoterpenes (8.02%). Among them, the content of (E,E)-α-Farnesene, (E)-nerolidol, (E)-β-ocimene, (Z)-dihydro-apofarnesol, linalool, and perillene were the main components. To the best of our knowledge, this study is the first to report the enantiomeric distribution of this species, reporting the terpenes (1R,5R)-(+)-α-pinene, (S)-(−)-limonene, (S)-(+)-Linalool, (1S,2R,6R,7R,8R)-(+)-α-copaene, (S)-(+)-(E)-Nerolidol, and (S)-(−)-(E)-Nerolidol. HS-SPME–GC has been effective in analyzing volatile compounds from *P. rubra* flowers, with chemical diversity influenced by harvest time. This study offers the first comprehensive and comparative aroma profile for the *P. rubra* species, which could be useful for future quality control.

## Figures and Tables

**Table 1:** Volatile chemical compounds of *Plumeria rubra* L. species detected using polar DB-5ms column.

N°	Compound	LRI ^a^	LRI ^b^	March % ± SD	May % ± SD	July % ± SD
1	Limonene	1019	1024	0.02		
2	(Z)-*β*-Ocimene	1027	1032	0.08	0.08	0.05
3	(E)-*β*-Ocimene	1038	1044	12.51 ± 0.24	10.82 ± 0.09	4.16± 0.15
4	Benzene acetaldehyde	1044	1036	0.07 ± 0.01	0.12 ± 0.02	0.09 ± 0.05
5	Linalool oxide <trans-> (furanoid)	1078	1084	0.04	0.1 ± 0.01	0.1 ± 0.02
6	Linalool	1103	1095	8.28 ± 0.16	11.05 ± 0.19	5.92 ± 0.15
7	trans-Vertocitral C	1110	1105		0.02 ± 0.01	
8	Perillene	1111	1102	5.48 ± 0.05	5.85	3.09 ± 0.06
9	1,3,8-ρ-Menthatriene	1123	1108	0.03	0.03 ± 0.01	
10	Phenyl ethyl alcohol	1124	1107			0.12 ± 0.05
11	2-Ethyl hexanoic acid	1131	1119			0.12 ± 0.02
12	Phenol <2-(1Z)-propenyl->	1133	1146	0.09	0.11	
13	Linalool oxide <trans-> (pyranoid)	1180	1173	0.02		0.03
14	(2E)-Hexenyl butanoate	1195	1193	0.02		
15	Methyl salicylate	1200	1190	0.03	0.05	
16	cis-4-Caranone	1214	1200	0.02		
17	iso-Dihydro carveol	1220	1212	0.14	0.21	0.05
18	NI	1231				0.03
19	(4Z)-Decen-1-ol	1265	1255	0.03 ± 0.01		
20	*α*-Ylangene	1374	1373	0.03	0.11 ± 0.01	
21	n-Tetradecane	1393	1400	0.04	0.02 ± 0.01	0.08
22	(Z)-*β*-Farnesene	1447	1440	0.23		0.23 ± 0.02
23	(E)-*β*-Farnesene	1450	1454		0.22 ± 0.13	
24	trans-Prenyl limonene	1460	1457	0.04 ± 0.01	0.07	
25	NI	1471				0.06
26	*γ*-Decalactone	1477	1465	0.03 ± 0.03	0.04 ± 0.02	0.1 ± 0.03
27	Cumacrene	1485	1470	1.11 ± 0.02	1.33 ± 0.01	1.01 ± 0.17
28	Widdra-2,4(14)-diene	1487	1481			0.04 ± 0.19
29	cis-Eudesma-6,11-diene	1491	1489		0.13 ± 0.03	
30	n-Pentadecane	1500	1500	0.05 ± 0.01	0.11 ± 0.01	0.05 ± 0.06
31	(E, E)-*α*-Farnesene	1505	1505	41.64± 0.65	40.87± 0.35	41.15± 0.29
32	*δ*-Decalactone	1506	1493			0.19 ± 0.28
33	*γ*-Patchoulene	1519	1502			0.05 ± 0.02
34	(Z)-*γ*-Bisabolene	1523	1514		0.11	
35	*β*-Sesquiphellandrene	1525	1521	0.02		
36	Decanediol <1,10->	1539	1547			0.03 ± 0.06
37	(Z)-Jasmolactone, extra C	1550	1566			0.11 ± 0.04
38	NI	1553				0.02
39	Geranyl butanoate	1554	1562	0.09 ± 0.04	0.15 ± 0.04	
40	(E)-Nerolidol	1565	1561	22.98 ± 0.61	21.4 ± 0.27	32.55 ± 1
41	(Z)-dihydro-Apofarnesol	1570	1571	6.46 ± 0.18	6.44 ± 0.12	9.89 ± 0.24
42	(3Z)-Hexenyl benzoate	1579	1565			0.04
43	*β*-Copaen-4-*α*-ol	1587	1590	0.06	0.06	0.11
44	n-Hexadecane	1600	1600			0.03 ± 0.02
45	Thujopsan-2-*β*-ol	1592	1588	0.05 ± 0.01	0.09 ± 0.01	0.03 ± 0.02
46	epi-Cedrol	1617	1618	0.16 ± 0.02		0.13 ± 0.02
47	Junenol	1618	1618		0.23	
48	Dill apiole	1628	1620			0.06
49	(Z)-Amyl cinnamaldehyde	1651	1647			0.03 ± 0.01
50	allo-Aromadendrene epoxide	1653	1639	0.02		
51	14-hydroxy-(Z)-Caryophyllene	1673	1666			0.02
52	Helifolenol B	1676	1677	0.03 ± 0.01	0.02 ± 0.01	
53	n-Tetradecanol	1677	1671			0.03
54	n-Heptadecane	1700	1700	0.05 ± 0.01	0.04 ± 0.01	0.05
55	Khusinol	1694	1679	0.02		0.03
56	(E)-Nerolidyl acetate	1714	1716			0.02
57	(2E,6Z)-Farnesal	1718	1713			0.03 ± 0.01
58	(2E,6E)-Farnesol	1738	1742	0.02 ± 0.01	0.03	
59	2-ethylhexyl-Salicylate	1806	1807	0.04 ± 0.01	0.06 ± 0.02	0.05 ± 0.01
Total identified (%)				100	100	99.87
Hydrocarbon sesquiterpenes (%)				43.12	42.95	42.53
Oxygenated sesquiterpenes (%)				23.38	21.89	32.95
Oxygenated monoterpenes (%)				14.03	17.28	9.51
Hydrocarbon monoterpenes (%)				12.73	11.04	4.21
Alcohols (%)				6.46	6.44	10.01
Aldehydes (%)				0.07	0.14	0.12
Esters (%)				0.12	0.20	0.06
Carboxylic acids (%)						0.12
NI						0.11

LRI ^b^: linear (arithmetic) calculated retention index; LRI ^a^: linear (arithmetic) retention index according to Adams [26]; % ± SD: area percentage and standard deviation of triplicate injections; NI: not identified.

**Table 2:** Volatile chemical compounds of *Plumeria rubra* L. species detected using polar HP-INNOWax column.

N°	Compound	LRI ^a^	LRI ^b^	March % ± SD	May % ± SD	July % ± SD	Reference
1	NI	1024				0.07	
2	*α*-Pinene	1026	1024	0.15	0.76	0.04	[27]
3	*α*-Thujene	1037	1037			0.14 ± 0.01	[28]
4	NI	1105			0.02		
5	n-Dodecane	1200	1200	0.02	0.02	0.02	[29]
6	(2E)-Hexenal	1221	1230	0.7	1.6		[30]
7	(E)-*β*-Ocimene	1246	1266	9.06± 1.20	4.96 ± 0.08	5.71± 0.09	[31]
8	5-Hepten-2-one, 6-methyl-	1331	1340	0.03	0.04	0.03	[30]
9	(3E)-Hexenol	1344	1352			0.11	[32]
10	1-Hexanol	1352	1369	0.82 ± 0.01	0.67 ± 0.01		[30]
11	2-Methylbutyl isovalerate	1300	1299	0.75	0.62		[33]
12	(E)-2-Hexen-1-ol	1350	1360	0.13	0.1		[34]
13	1,3,8-ρ-Menthatriene	1405	1438	0.08		0.07 ± 0.01	[35]
14	*α*-Copaene	1410	1458	0.03	0.03		[28]
15	Perillene	1422	1425	3.19 ± 0.01	1.83 ± 0.03	3.00 ± 0.08	[36]
16	1-Octen-3-ol	1425	1462	0.02	0.02		[30]
17	cis-Linalool oxide, furanoid	1431	1437	0.02	0.06		[37]
18	trans-Linalool oxide, (furanoid)	1449	1446	0.21	0.54	0.16	[28]
19	Heptadecane, 2,6,10,15-tetramethyl-	1469	1660	0.09	0.08		[38]
20	Cumacrene	1488	1472	1.04 ± 0.01	1.1 ± 0.02	0.93 ± 0.02	[39]
21	n-Pentadecane	1500	1500	0.18	0.15	0.16	[40]
22	α-Yanglene	1503	1493			0.52 ± 0.02	[37]
23	benzaldehyde	1506	1518	0.02	0.07		[28]
24	NI	1536			0.03		
25	Linalool	1538	1543	7.8 ± 0.04	4.93 ± 0.04	3.27 ± 0.74	[28]
26	NI	1539				0.19 ± 0.07	
27	Hexadecane <n->	1579	1599	0.05	0.04 ± 0.02		[41]
28	5-methylfurfural	1580	1608	0.02			[42]
29	Prenyl limonene <trans->	1595				0.66 ± 0.02	[43]
30	Guaiol acetate	1610		0.18	0.24 ± 0.01		
31	(Z)-*β*-Farnesene	1633	1668	0.22	0.32 ± 0.01	0.23 ± 0.02	[31]
32	Myrtenal	1643	1646			0.51	[37]
33	Heptadecane	1700	1704	0.13	0.15		[44]
34	NI	1705			0.13		
35	(E, E)-*α*-Farnesene	1740	1758	38.48 ± 0.04	38± 0.19	50.59 ± 0.59	[31]
36	δ-cadinene	1743	1764	0.09	0.1		[37]
37	NI	1744			0.12 ± 0.01		
38	trans-Linalool oxide (pyran)	1751	1749	0.09	0.11		[45]
39	NI	1762				0.07 ± 0.01	
40	Myrtenol	1811	1804	0.03	0.06	0.02	[37]
41	trans-Geranylacetone	1844	1867		0.08	0.04	[46]
42	NI	1869		0.05	0.06	0.02	
43	NI	1892		0.05			
44	2-Ethyl hexanoic acid	1923	1950	0.08	0.07	0.03	[47]
45	Phenylethyl Alcohol	1922	1872	0.08	0.16	0.43 ± 0.05	[44]
46	NI	1933		0.10	0.08	0.04	
47	NI	2011			0.12	0.07	
48	Aromadrene epoxide (allo)	2029	2046	0.07		0.61 ± 0.03	[40]
49	NI	2030		0.08	0.22		
50	NI	2035		0.08	0.07	0.04	
51	(E)-Nerolidol	2058	2036	26.28 ± 0.07	33.02 ± 2.11	23.16 ± 0.15	[28]
52	*δ*-Octalactone	2085	1967			0.06	[48]
53	(Z)-dihydro-Apofarnesol	2124	2137	7.58 ± 0.03	7.7 ± 0.13	8.62 ± 1.58	[49]
Total identified (%)				97.96	97.78	99.62	
Hydrocarbon sesquiterpenes (%)				40.00	39.75	52.57	
Oxygenated sesquiterpenes (%)				26.40	33.21	23.81	
Oxygenated monoterpenes (%)				11.20	7.30	6.45	
Hydrocarbon monoterpenes (%)				9.30	4.96	5.92	
Alcohols (%)				8.64	8.83	9.12	
Aldehydes (%)				0.07	2.43		
NI				2.04	2.33	0.38	

LRI ^b^: linear (arithmetic) calculated retention index; LRI ^a^: linear (arithmetic) retention index according to references; % ± SD: area percentage and standard deviation of triplicate injections; NI: not identified.

**Table 3:** Chemical structure, odor, and biological properties of the major compounds identified.

Compounds	Odor	Biological Properties	Ref.
(E,E)-*α*-Farnesene 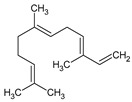	Floral—green apple	Antimicrobial and antifungal activities.	[50]
(E)-Nerolidol 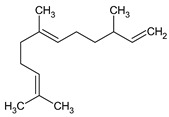	Woody	Antioxidant, antifungal, and antimicrobial activity, gastroprotective, cytotoxic.	[51,52]
(E)-*β*-Ocimene 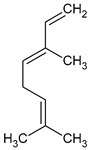	Sweet, herbal	Anti-inflammatory, antibiotic, and antioxidant activities.	[53,54]
Linalool 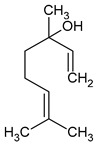	Floral, lavender-like	Antioxidant and antibacterial activity.	[53,55,56]
(Z)-dihydro-apofarnesol 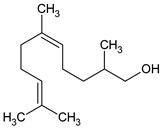	Not reported	Not reported	
Perillene 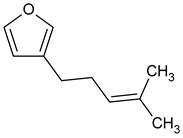	Not reported	Not reported	

**Table 4:** Enantiomeric distribution of *Plumeria rubra* L. flowers from Ecuador on β-cyclodextrin column.

Enantiomeric Compounds	RI ^c^	Distribution %	*e.e* %
(1R,5R)-(+)-α-pinene	924 ± 0.9	100	100
(S)-(−)-limonene	1051 ± 0.7	100	100
(S)-(+)-Linalool	1179 ± 0.6	100	100
(1S,2R,6R,7R,8R)-(+)-α-copaene	1322 ± 0.02	100	100
(S)-(+)-(E)-nerolidol	1683 ± 1.2	99.85	99.7
(R)-(−)-(E)-nerolidol	1701 ± 1.7	0.14	

RI ^c^: calculated retention index; *e.e*: enantiomeric excess; SD: standard distribution.

## Data Availability

The original contributions presented in the study are included in the article, further inquiries can be directed to the corresponding author.
